# Supplementary material for: FERN – a Java framework for stochastic simulation and evaluation of reaction networks
Source: BMC Bioinformatics. 2008 Aug 29;9:356. doi: 10.1186/1471-2105-9-356 (PMC2553347; doi:10.1186/1471-2105-9-356)
Supplement: Additional file 1 — FERN distribution, Version 1.3. This archive contains the FERN source code and binaries as well as documentation and example models in FernML and SBML. [file 1471-2105-9-356-S1.zip › fern/doc/javadoc/fern/network/sbml/package-summary.html]

fern.network.sbml


---


|  |  |  |
| --- | --- | --- |
| **Overview** \| **Package** \| Class \| **Use** \| **Tree** \| **Deprecated** \| **Index** \| **Help** | |  |
| **PREV PACKAGE**   **NEXT PACKAGE** | **FRAMES**    **NO FRAMES**     **All Classes** |  |


---

## Package fern.network.sbml

Provides the classes for parsing and using sbml based networks.

**See:**
  
          **Description**

| **Class Summary** | |
| --- | --- |
| **MathTree** | Representation of am evaluation tree. |
| **MathTree.ConstLeaf** |  |
| **MathTree.GlobalLeaf** |  |
| **MathTree.InnerNode** |  |
| **MathTree.Node** |  |
| **MathTree.VarLeaf** |  |
| **SBMLEventHandlerObserver** | Observer which handles an event of a sbml model. |
| **SBMLNetwork** | For specifications of the sbml format refer to http:\\www.sbml.org. |
| **SBMLPropensityCalculator** | Propensity calculator which is used for `SBMLNetwork`s. |

## Package fern.network.sbml Description

Provides the classes for parsing and using sbml based networks.

---


|  |  |  |
| --- | --- | --- |
| **Overview** \| **Package** \| Class \| **Use** \| **Tree** \| **Deprecated** \| **Index** \| **Help** | |  |
| **PREV PACKAGE**   **NEXT PACKAGE** | **FRAMES**    **NO FRAMES**     **All Classes** |  |


---
